# Impaired CD8^+^ T cell responses upon Toll-like receptor activation in common variable immunodeficiency

**DOI:** 10.1186/s12967-016-0900-2

**Published:** 2016-05-17

**Authors:** Camila de Lollo, Dewton de Moraes Vasconcelos, Luanda Mara da Silva Oliveira, Tiago de Oliveira Titz, Magda Carneiro-Sampaio, Cristina Miuki Abe Jacob, Alberto José da Silva Duarte, Maria Notomi Sato

**Affiliations:** Laboratory of Dermatology and Immunodeficiencies, LIM-56, Department of Dermatology, Medical School, Tropical Medicine Institute, University of São Paulo, Av. Dr. Enéas de Carvalho Aguiar, 500, 3rd Floor, São Paulo, 05403-000 Brazil; Department of Pediatrics, Medical School, University of São Paulo, São Paulo, Brazil

**Keywords:** Common variable immunodeficiency, Exhaustion and activation markers, Toll-like receptor agonists, Tc22/Th22, Polyfunctional T cells

## Abstract

**Background:**

Infections caused by bacteria or viruses are frequent in common variable immunodeficiency (CVID) patients due to antibody deficiencies, which may be associated with altered T cell function. CVID patients are frequently in contact with pathogen-associated molecular patterns (PAMPs), leading to the activation of innate immunity through Toll-like receptors (TLR) affecting T cell activation. We evaluated the effect of TLR activation on T cells in CVID patients undergoing intravenous immunoglobulin (IVIg) replacement using synthetic ligands.

**Methods:**

Expression of exhaustion, activation and maturation markers on T cells from peripheral blood as well as regulatory T cells and follicular T cells in peripheral blood mononuclear cells (PBMCs) from CVID and healthy individuals were evaluated by flow cytometry. PBMCs cultured with TLR agonists were assessed for intracellular IFN-γ, TNF, IL-10, IL-17a or IL-22 secretion as monofunctional or polyfunctional T cells (simultaneous cytokine secretion) by flow cytometry.

**Results:**

We found increased expression of the exhaustion marker PD-1 on effector memory CD4^+^ T cells (CD45RA^−^CCR7^−^) in the peripheral blood and increased expression of CD38 in terminally differentiated CD8^+^ T cells (CD45RA^+^CCR7^−^). Furthermore, a decreased frequency of naïve regulatory T cells (CD45RA^+^Foxp3^low^), but not of activated regulatory T cells (CD45RA^−^Foxp3^high^) was detected in CVID patients with splenomegaly, the non-infectious manifestation in this CVID cohort (43.7 %). Moreover, the frequency of peripheral blood follicular helper T cells (CD3^+^CD4^+^CXCR5^+^PD-1^+^ICOS^+^) was similar between the CVID and control groups. Upon in vitro TLR3 activation, a decreased frequency of CD8^+^ T cells secreting IFN-γ, IL-17a or IL-22 was detected in the CVID group compared to the control group. However, a TLR7/TLR8 agonist and staphylococcal enterotoxin B induced an increased Th22/Tc22 (IL-22^+^, IFN-γ^−^, IL-17a^−^) response in CVID patients. Both TLR2 and TLR7/8/CL097 activation induced an increased response of CD4^+^ T cells secreting three cytokines (IL-17a, IL-22 and TNF)in CVID patients, whereas CD8^+^ T cells were unresponsive to these stimuli.

**Conclusion:**

The data show that despite the unresponsive profile of CD8^+^ T cells to TLR activation, CD4^+^ T cells and Tc22/Th22 cells are responsive, suggesting that activation of innate immunity by TLRs could be a strategy to stimulate CD4^+^ T cells in CVID.

**Electronic supplementary material:**

The online version of this article (doi:10.1186/s12967-016-0900-2) contains supplementary material, which is available to authorized users.

## Background

Common variable immunodeficiency (CVID) is a primary immunodeficiency characterized by susceptibility to recurrent infections due to inadequate quantity and quality of protective antibodies [1]. There is an immense phenotypic heterogeneity of patients with CVID, which can be observed in the variability of the age at onset of disease and related comorbidities, including autoimmune diseases, malignancies, chronic lung diseases, splenomegaly and gastrointestinal diseases [2, 3]. The current treatment consists of intravenous IgG (IVIG) replacement every 3–4 weeks [4] with the goal of protecting patients against infection by extracellular pathogens.

Relapsing bacterial/viral infections in CVID trigger strong stimulation of innate immunity. Upon pathogen-associated molecular pattern (PAMP) expression, pattern recognition receptors (PRRs), including Toll-like receptors (TLRs), trigger intracellular signals to innate immune cells to contain the infection and return to homeostasis [5]. However, defective TLR7 and TLR9 signaling in B cells and plasmacytoid dendritic cells (pDCs), deficient IFN-α secretion, and impaired B cell function alter innate immune responses in CVID, thus preventing TLR-mediated enhancement of humoral immunity in vivo and possibly increasing susceptibility to enteroviral and rhinoviral infections [6, 7].

Moreover, there are several CD4^+^ T cell abnormalities and functional alterations in CVID, such as a reduction in CD4^+^ T cell counts, reduced proliferation capacity and/or impaired cytokine production [2, 8–10]. Bacteria-specific CD4^+^ T cells from CVID patients are functionally impaired and express high levels of programmed death 1 (PD-1) [11]. Intravenous immunoglobulin (IVIg) treatment significantly reduces endotoxemia and PD-1^+^ expression on CD4^+^ T cells, restoring bacteria-specific CD4^+^ T cell cytokine production and proliferation [11] and reducing CD8^+^ T cell activation [12].

Antigen-presenting cells have been the primary focus of TLR research, although TLRs are also expressed on cells of the adaptive immune system, such as T and B cells. Activation of TCR up-regulates TLR expression, rendering these T cells responsive to TLR agonists, such as TLR5 and TLR7/TLR8, which demonstrate co-stimulatory capacity [13, 14]. It has been suggested that TLR stimulation of T cells can function as a co-stimulator during the initiation of an adaptive immune response to aid the survival of memory T cells, as well as in the rapid induction of a memory response [15]. The T cell perturbations in CVID can be attenuated by IVIg treatment, whereas the adjuvant role of TLR activation, which directly or indirectly influences CD4/CD8 T cell responses, could be of great interest.

In the present study, the results show the adjuvant role of TLR agonists in adaptive immunity, leading to a polyfunctional response primarily by CD4^+^ T cells and to increased Th22/Tc22 responses in CVID.

## Methods

### Patients

CVID patients were followed at the primary immunodeficiency diseases outpatient clinic (ADEE3003) of the Hospital das Clínicas, Faculdade de Medicina da Universidade de São Paulo (HC/FMUSP). The CVID group (n = 16) consisted of 10 males and six females (aged 22–65), and the healthy donors (n = 16) consisted of seven males and nine females (aged 21–65). All CVID patients fulfilled the European society for immunodeficiencies (ESID) and Pan-American Group for Immunodeficiency (PAGID) criteria [16], and all were on IVIg replacement at a dose of 400–800 mg/kg/3–4 weeks (to maintain trough levels above 8.0 g/L). Blood samples were collected before IVIg infusion; during this period, all patients were free of acute or chronic infections, and none was receiving corticosteroid therapy. The research involving human participants reported in this study was approved by the São Paulo University Institutional Use Committee. Study participants were enrolled after signing and dating an approved informed consent. Our Ethics Committee approved the consent procedure. The approval number is (0295/09).

### Flow cytometry in peripheral blood

To analyze the exhaustion/activation markers at different stages of T cell maturation (naïve CCR7^+^ CD45RA^+^, effector CCR7^−^ CD45RA^−^, memory CCR7^+^ CD45RA^−^, memory RA CCR7^−^ CD45RA^+^) in the peripheral blood, venous blood was collected in EDTA-anticoagulated tubes, and staining was performed using the following antibodies: CD3 BV605 (SK7), CD4 V500 (RPA-T4), CD8 PerCP-Cy5.5 (RPA-T8), CD45RA APC-H7 (HI100), CD127 PE-Cy7 (HIL-7R-M21), CCR7 APC (3D12) and PD-1 PE (MIH4). To analyze follicular helper T cells, CD3 BV605 (SK7), CD4 V500 (RPA-T4), CXCR5 PerCP-Cy5.5 (RF8B2) and PD-1 FITC (MIH4) were evaluated. Antibodies were purchased from BD Pharmingen (San Jose, CA, USA). Approximately 70 µL of whole blood was stained for 20 min and then incubated for 15 min with FACS lysing solution (BD FACS Lysing; BD Biosciences, San Jose, CA) to lyse the erythrocytes. After two washes in an isotonic solution (Hemoton SPEC; Brazil), 300,000 events were acquired using a flow cytometer (LSR Fortessa; BD Biosciences) and were analyzed using FlowJo Software (Tree Star, Ashland, OR, USA).Regulatory T cells were evaluated according to the instructions for the FoxP3 staining kit (eBiosciences, San Diego, CA, USA).

### Flow cytometry of in vitro TLR-activated cells

Peripheral blood mononuclear cells (PBMCs) were isolated by Ficoll-Hypaque density-gradient centrifugation (Amersham Pharmacia Biotech, NJ, USA) and were resuspended in RPMI 1640 (Gibco Invitrogen, Carlsbad, CA, USA) after two washes in medium supplemented with gentamicin (10 µg/mL) and 10 % AB human serum (Sigma-Aldrich, St Louis, MO, USA). PBMCs at 1.5 × 10^6^ cells/mL were incubated in 48-well plates (Costar, Cambridge, MA, USA) with medium or with 5 µg/mL TLR2 agonist (Pam3CSK4), 40 ng/mL TLR3 (Poly-RIG) agonist or 5 µg/mL TLR7/8 (CL097) agonist (InvivoGen, San Diego, CA, USA) for 20 h or 1 µg/mL *Staphylococcus aureus* enterotoxin B (SEB, Sigma-Aldrich), 10 ng/mL phorbol myristate acetate (PMA) (Sigma-Aldrich) and 1 µg/mL ionomycin (Iono) (Sigma-Aldrich) for 6 h at 37 °C in 5 % CO_2_. Brefeldin A (10 µg/mL, Sigma) was added to the cultures for the last 4 h. PBMC cultures were washed and incubated with LIVE/DEAD Fixable Red Dead Cell Stain Kit (Invitrogen, Carlsbad, CA, USA) for 30 min at room temperature, followed by fixation with Cytofix/Cytoperm solution (BD Bioscience) for 20 min and permeabilization with Perm/Wash solution for 20 min at 4 °C. The cells were then stained with CD3 BV605 (SK7), CD4 V500 (RPA-T4), CD8 PerCP-Cy5.5 (RPA-T8), CD38 Alexa Fluor 700 (HIT2), IFN-γ V450 (B27), TNF Pe-Cy7 (Mab11), IL-10 APC (JES3-19F1), IL-17a Alexa Fluor 488 (eBiosciences) and IL-22 PE, (eBiosciences); unless otherwise mentioned, all antibodies were purchased from BD Biosciences (San Jose, CA, USA). Next, the samples were washed with Perm/Wash buffer (BD Biosciences) and diluted in isotonic solution. A total of 500,000 events were acquired and analyzed by flow cytometry (LSR Fortessa, BD Biosciences, USA) using the FACS-Diva (BD Bioscience) and FlowJo10.0.6 (Tree Star, Ashland, OR, USA) software programs. Fluorescence Minus One (FMO) controls were performed for all antibody panels to check proper compensation and to define positive signals. Boolean gate arrays were created using FlowJo software. These analyses determined the expression frequency of each cytokine based on all possible combinations of the five cytokines. Polychromatic flow cytometry data were analyzed with the SPICE Program (Version 2.9, Vaccine Research Center, NIAID, USA).

### Statistical analysis

All cytokine measurements were background-subtracted, taking into account the frequency of cells producing cytokines in the absence of antigenic stimulation. The nonparametric Mann–Whitney test was used to compare variables of CVID and healthy controls. The comparison of the three groups healthy individuals (HC) versus CVID with and without splenomegaly was performed by Kruskal–Wallis test followed by Dunn’s multiple comparisons test. *P* ≤ 0.05 was considered statistically significant.

## Results

### Exhaustion/activation T cell markers and frequency of effector/regulatory T cells in CVID

To evaluate whether the activation of innate immunity via TLR activation could enhance the adaptive response, we previously evaluated the activation/exhaustion profiles of CD4^+^ and CD8^+^ T cells. Moreover, considering that IVIg treatment partially restores CD4^+^ T cell activation [17], we evaluated the markers related to exhaustion (programmed cell death, CD279, PD-1), resting/naïve status (interleukin (IL)-7 receptor alpha chain (CD127), and activation (CD38) at different stages of T cell maturation as well as in regulatory T cells in the CVID and HC groups. The follicular T cells (CD4^+^ CXCR5^+^ PD-1^+^ ICOS^+^), which are specialized providers of T cell help to B cells and are essential for germinal center formation, were also evaluated in peripheral blood from the CVID and HC groups.

**Fig. 1:**
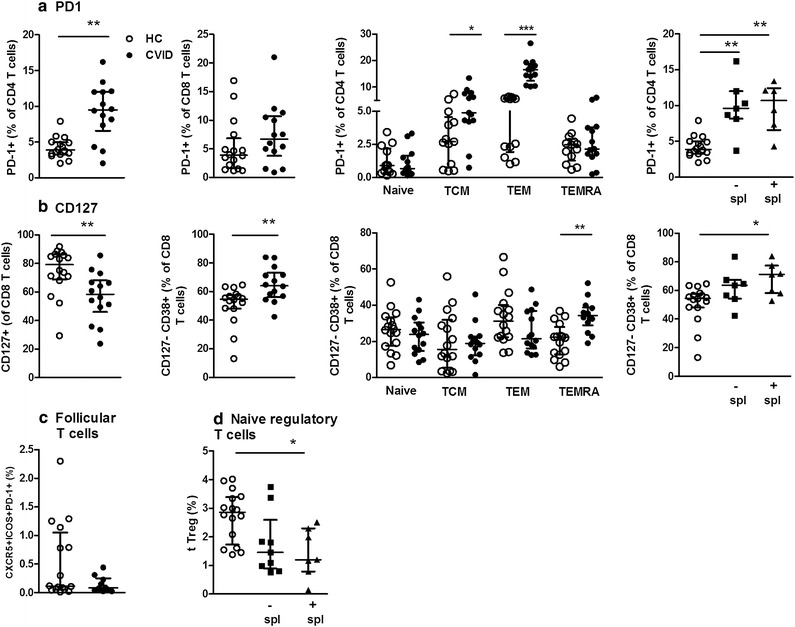
Altered exhaustion/activation marker expression on T cells and frequency of regulatory T cells in CVID. PBMCs from healthy control (HC, n = 16) and CVID patients (n = 14) were assessed for (**a**) PD-1 and (**b**) CD127 expression in CD4^+^ and CD8^+^ T cells, according to the maturation stage, and for the presence (+) or absence (−) of splenomegaly (spl); (**c**) follicular T cells (CD3^+^ CD4^+^ CXCR5^+^ PD-1^+^ ICOS^+^) and (**d**) naïve regulatory T cells (CD3^+^ CD4^+^ CD45RA^+^ Foxp3^+^) were determined by flow cytometry. Data represent the median and the interquartile range. *p ≤ 0.05, **p ≤ 0.001, ***p ≤ 0.0001

In the present study, 16 CVID patients and 16 HC were enrolled (Table 1). The percentage of B cells in the CVID patients was decreased compared to that in the HC group, with increased frequencies of naïve B cells and decreased memory B cells. Figure 1a shows an increased frequency of CD4^+^ PD-1^+^ T cells in CVID patients compared to HC individuals, whereas no difference was observed in CD8^+^ T cells. Evaluating CD4^+^ PD-1^+^ T cells according to T cell maturation stage, we found an increased frequency of memory T cells in CVID patients, including the central memory (TCM) (CD45RA^−^ CCR7^+^) and effector memory (TEM) stages (CD45RA^−^ CCR7^−^). Considering the several clinical features frequently observed in CVID subjects and the fact that the factors that induce splenomegaly are not well known, we compared the immunologic cellular markers of CVID patients with and without splenomegaly; however, we did not observe any differences between the groups.

**Table 1:** Demographic data and laboratory characteristics of studied CVID subjects

Patients CVID	CVID total (n = 16)	HC total (n = 16)
Median age^a^	34.0 (22–65)	36 (21–65)
Gender (male/female)	10/6	7/9
Onset of disease (early/late)^b^	9/7	
Autoimmunity manifestations (%)	19	
Bronchiectasis (%)	62.5	
Splenomegaly (%)	43.75	
Lymphadenopathy (%)	1.5	
GI symptoms (%)	25	
Granulomas (%)	12.5	
Cancer (%)	12.5	
Median duration of IVIg (years)	9.0 (3–15)	
Immunoglobulin G (mg/dL)^c^	771.5 (459–926)	
Immunoglobulin A (mg/dL)	5 (0.2–29.4)	
Immunoglobulin M (mg/dL)	10.2 (1.85–32.4)	
B cells (CD3− CD19+) (%)	6.6 (4.58–10.13)	30.4 (9.68–40.98)
Naive B cells (IgD+ CD27−) (%)	88.9 (75.78–93.60)	36.65 (31.05–57.50)
Memory B cells (IgD− CD27+) (%)	1.63 (0.4–3.4)	17.75 (11.25–21.20)

^a^Values are expressed as median with interquartile range

^b^Early onset was determined before 20 years-old and late onset after 20 years-old

^c^IgG levels during the IVIg treatment

Moreover, decreased CD8^+^ CD127^+^ T cell percentages but not CD4^+^ T cell percentages (data not shown) were observed in CVID patients compared to HC (Fig. 1b). As expected in CVID, an increased frequency of activated CD8^+^ CD127^−^ CD38^+^ T cells was verified in the terminally differentiated effector memory (TEMRA) subset in CVID individuals with splenomegaly.

The frequency of peripheral blood follicular helper T cells (Tfh) (CD3^+^ CD4^+^ CXCR5^+^ PD-1^+^ ICOS^+^) was similar between the HC and CVID groups (Fig. 1c). All CVID patients expressed ICOS.

The reduced frequency of Tregs in CVID patients and its association with autoimmunity as well as immune dysregulation has been previously reported [3, 18]. Thus, we analyzed the subpopulation of Tregs in CVID. The Treg cells have been subgrouped in three distinct subpopulations as (i) CD45RA^+^ Foxp3^low^ naïve Treg cells (nTreg cells), (ii) CD45RA^−^ Foxp3^high^ activated Treg cells (aTreg cells), both of which are suppressive in vitro, and non-suppressive cytokine-secreting CD45RA^−^ Foxp3^low^ T cells (non-Treg cells) [19, 20]. We found a decreased frequency of naïve Treg cells (CD45RA^+^ Foxp3^low^) in CVID patients compared to HC individuals. In addition, the decreased frequency of Treg cells was associated with splenomegaly in CVID patients compared to the HC group (Fig. 1d).

Collectively, these results show the presence of exhaustion markers on CD4^+^ T cells, confirming previous data [17], and indicate that these markers are mainly expressed on memory subsets, such as TCM and TEM. As TCM cells can differentiate into TEM cells, the PD-1 on these subsets suggests the exhaustion of effector TCD4^+^ function. Additionally, CD8^+^ CD38^+^ T cells, which chronically activate TEMRA cells, and decreased CD127^+^ may, together, indicate an immunosenescence of CD8^+^ T cells in CVID. Moreover, Treg cell levels are reduced in CVID patients [17, 21], which we found to be associated with the naïve Treg subset, not the inducible subset at the periphery.

### Impaired TLR responses by CD8^+^ T cells in CVID

Infections caused by viruses or bacteria are common features in CVID. Because TLR stimulation using synthetic/natural ligands may mimic extracellular and intracellular pathogens, we analyzed the effect of TLR activation by ligands on the secretion of cytokines by T cells. We assessed TLR2/Pam3CSK4, TLR3/Poly-RIG and TLR7/8/CL097 agonists and used PMA/ionomycin as a nonspecific activating control. The gating strategy is shown in Additional file 1.

**Fig. 2:**
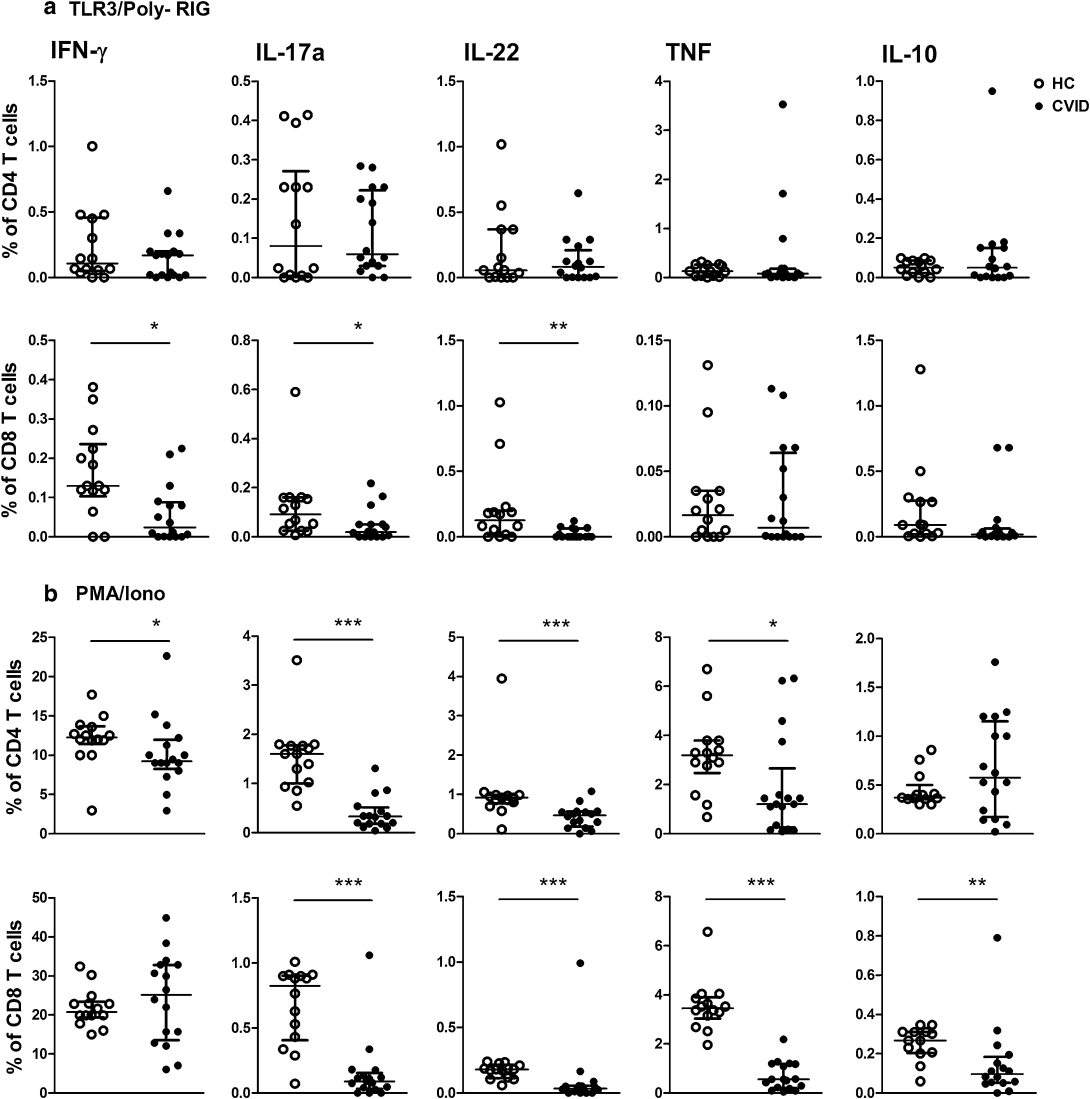
Altered cytokine response by CD8^+^ T cells upon TLR3 stimulation and in response to PMA/ionomycin in CVID. PBMCs from healthy controls (HC, n = 16) and CVID patients (n = 16) were cultivated with medium (baseline) or (**a**) TLR-3 agonist (Poly-RIG) for 20 h or (**b**) PMA/ionomycin for 6 h. CD4^+^ and CD8^+^ T cells secreting IFN-γ, IL-17a, IL-22, TNF and IL-10 were assessed by flow cytometry. Frequencies of stimulated CD4^+^ CD8^+^ T cells were subtracted from baseline values. Data represent the median and the interquartile range. *p ≤ 0.05, **p ≤ 0.005, ***p ≤ 0.0001

Figure 2 shows that upon TLR3 activation, IFN-γ, IL-17a, IL-22, TNF or IL-10 secretion was observed in CD4^+^ T cells at similar levels in the CVID and HC groups. In contrast, a decreased frequency of CD8^+^ T cells secreting IFN-γ, IL-17a or IL-22 was detected in the CVID group (Fig. 2a). Activation by PMA/ionomycin induced impaired IFN-γ, IL-17a, IL-22 and TNF but not IL-10 responses by CD4^+^ T cells in CVID compared to HC. Similarly, the CD8^+^ T cell cytokine response was decreased, including IL-10 secretion, in CVID patients (Fig. 2b).

These data show that CD8^+^ T cells are down-modulated by TLR3 stimulation. This finding indicates an impairment of CD8^+^ T cells in CVID, as TLR3 ligand is able to stimulate fully functional memory CD8 T cells in the absence of CD4^+^ T cells [22].

#### High frequency of circulating Th22/Tc22 upon TLR and SEB stimulation in CVID

The strategy to evaluate the secretion of IL-22, without the exclusion of other cytokines, includes other populations, such as Th17. We therefore analyzed excluding IFN-γ and IL-17 to consider Th22/Tc22 cells [23, 24] in the CVID and HC groups. The gating strategy for Th22 and Tc22 is shown in Additional file 2.

**Fig. 3:**
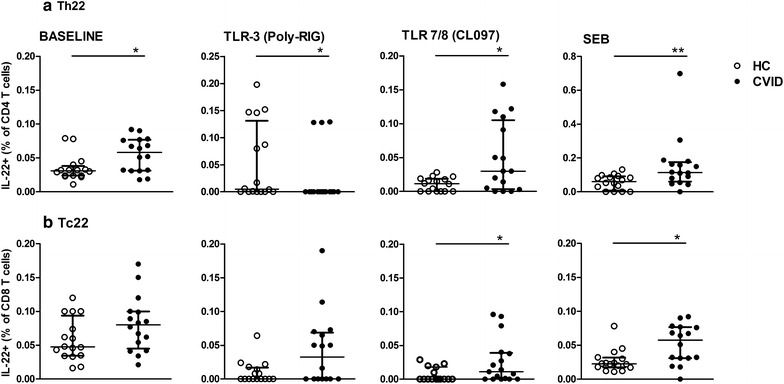
Presence of circulating Th22 and Tc22 cells in CVID patients. PBMCs from healthy controls (HC, n = 16) and CVID patients (n = 16) were cultivated with medium (baseline) or agonists of TLR3 (Poly-RIG) and TLR7/8 (CL097) for 20 h or SEB for 6 h and assessed by flow cytometry. **a** Th22 and **b** Tc22 cell frequencies were obtained by excluding IFN-γ and IL-17a. Data represent the median and the interquartile range. *p ≤ 0.05, **p ≤ 0.01

Interestingly, we detected an increased baseline Th22 frequency in CVID in comparison to HC (Fig. 3a). In addition, both Th22/Tc22 from CVID patients had an increased response to TLR7/8/CL097 agonists as well as to SEB (Fig. 3b); however, Th22 cells were decreased in CVID compared to HC subjects upon TLR3 activation. The strategy of Th22/Th22 evaluation may represent a different population due to the different results after TLR activation.

#### Polyfunctional CD4^+^ and CD8^+^ T cell responses to TLR activation in CVID

Polyfunctional T cells can provide more effective immune responses to pathogens than single cytokine-producing cells. We next evaluated the ability of cells to secrete IFN-γ, IL-10, IL-17a, IL-22 and TNF upon TLR, PMA/ionomycin, or SEB stimulation. The gating strategy to detect simultaneous cytokine secretion in CD4^+^ and CD8^+^ T cells is shown in Additional file 3. Because a Boolean strategy generates several combinations, we present only the data with significant differences.

**Fig. 4:**
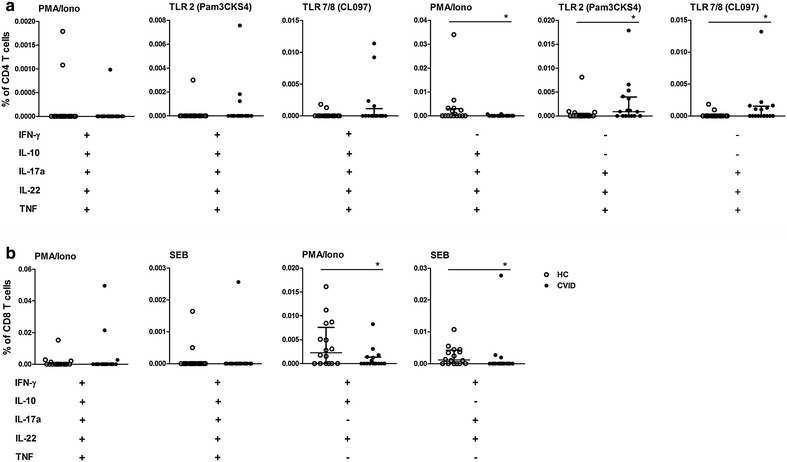
Activation by TLR agonists induces polyfunctional CD4^+^ T cells in CVID. The combinations of IFN-γ, IL-10, IL-17a, IL-22 and TNF secreted by (**a**) CD4^+^ and (**b**) CD8^+^ T cells induced by TLR2 (Pam3CSK4), TLR7/8 (CL097), PMA/ionomycin or SEB from HC (n = 16) and CVID (n = 16) individuals were derived from a Boolean strategy. Frequencies of stimulated CD4^+^ or CD8^+^ T cells were subtracted from baseline values. Data represent the median and the interquartile range. *p ≤ 0.05

Overall, CD4^+^ T cells simultaneously secreting 4–5 cytokines stimulated by TLR agonists or PMA/ionomycin were preserved in CVID (Fig. 4). The evaluation of polyfunctional cells secreting three cytokines indicated an increased frequency of CD4^+^ T cells secreting IL-17a, IL-22 and TNF induced by TLR7/8/CL097 and TLR2/Pam3CKS4 in CVID compared to HC individuals. Similar to monofunctional CD4^+^ T cells activated by PMA/ionomycin, a decreased percentage of CD4^+^ T cells secreting four cytokines (IL-10, IL-17a, IL-22 and TNF) was also observed in CVID upon PMA/ionomycin stimulation.

Polyfunctional cells secreting five cytokines were balanced in both groups, whereas CD8^+^ T cells secreting three cytokines in CVID were decreased in response to PMA/ionomycin (IFN-γ, IL-10, IL-22) as well as SEB (IFN-γ, IL-17a, IL-22) (Fig. 4b). These data show that TLR activation induces polyfunctionality only in CD4^+^ T cells in CVID, and there is a disturbed PMA/ionomycin response by both CD4^+^ and CD8^+^ T cells.

**Fig. 5:**
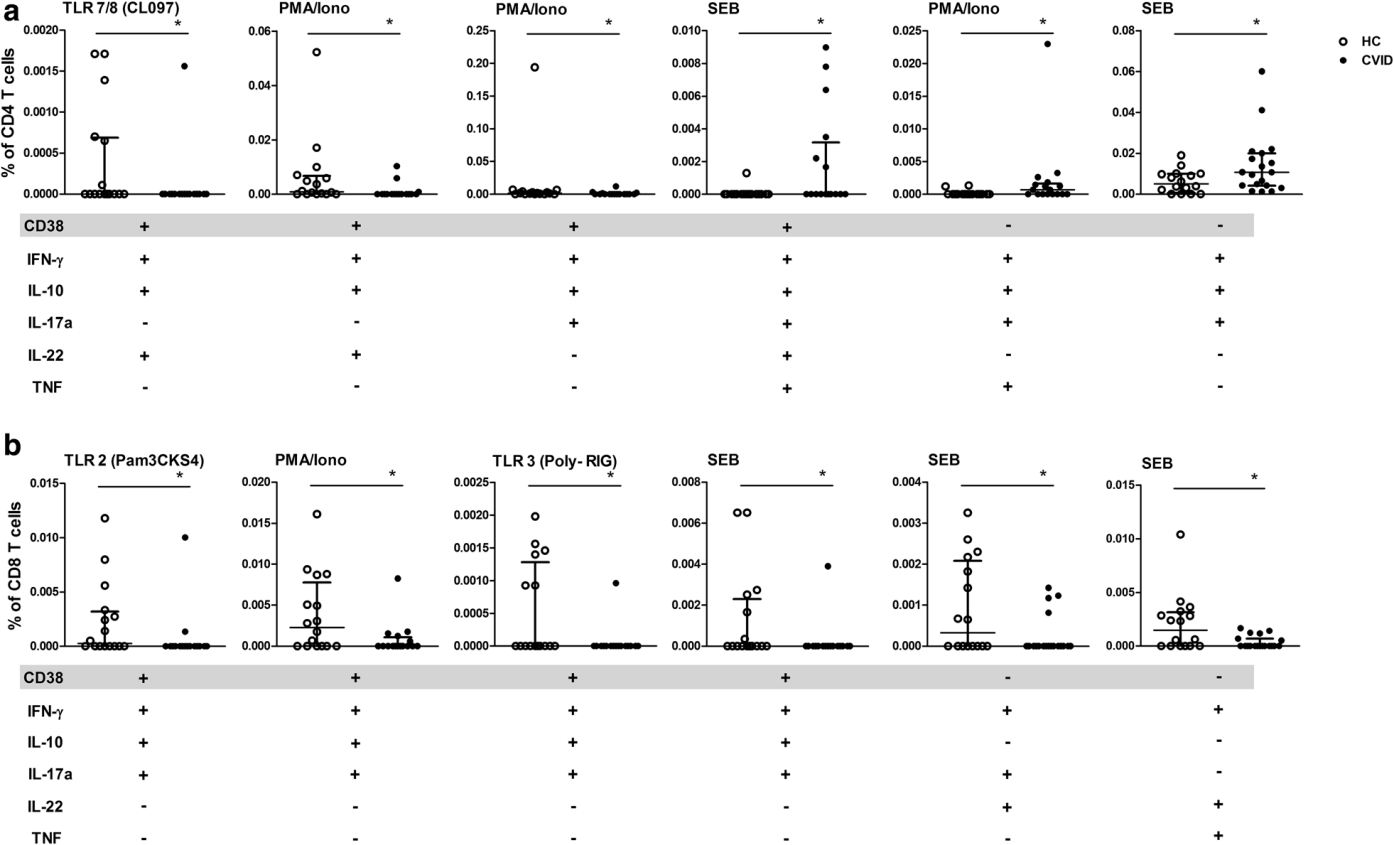
Impaired CD38^−^ or CD38^+^ CD8^+^ T cell polyfunctionality induced by SEB in CVID. The combinations of IFN-γ, IL-10, IL-17a, IL-22, and TNF secreted by (**a**) CD4^+^ and (**b**) CD8^+^ T cells in the presence or absence of CD38 expression, induced by TLR2 (Pam3CSK4), TLR3 (Poly-RIG), TLR7/8 (CL097), PMA/ionomycin or SEB, from HC (n = 16) and CVID (n = 16) individuals were evaluated by a Boolean strategy. Frequencies of stimulated CD4^+^ or CD8^+^ T cells were subtracted from baseline values. Data represent the median and the interquartile range. *p ≤ 0.05

To further analyze T cell activation, we evaluated polyfunctionality (IFN-γ, IL-10, IL-17a, IL-22 and TNF) in CD38^+^ T cells. The gating strategy is shown in Additional file 3. Figure 5a shows that CD38^+^ CD4^+^ T cells had decreased secretion of 4-cytokine combinations in CVID, whether induced by TLR7/8-CL097 or PMA/ionomycin. Polyfunctional CD38^+^ CD4^+^ T cells secreting five cytokines (IFN-γ, IL-17a, IL-22, TNF and IL-10) in response to SEB were observed in CVID. Curiously, among CD4^+^ CD38^−^ T cells, the PMA response for 4-cytokine combinations (IFN-γ, IL-10, IL-17a, and TNF) was maintained in CVID. The decreased polyfunctional response to TLR activation by CD8^+^ T cells was found in both CD38^+^and CD38^−^ T cell subsets (Fig. 5b). These data showed that CD4^+^CD38^−^ T cells induced by PMA/ionomycin are able to simultaneously secrete four cytokines (IFN-γ, IL-17a, IL-22, and TNF) in CVID, whereas polyfunctionality was decreased in CD8^+^ T cells.

Our findings show that monofunctional or polyfunctional cytokines by CD8^+^ T cells are impaired upon innate stimulation in CVID, in contrast to the preserved function of polyfunctional CD4^+^ T cells.

## Discussion

CVID patients present recurrent bacterial infections, and a significant proportion develop autoimmune, inflammatory or lympho proliferative complications. The activation of innate immunity through TLRs by synthetic ligands may mimic PAMPs from bacteria or viruses, and these effects on T cells have not been well explored. Our data confirm the up-regulation of activation/exhaustion markers in T cells in CVID, occurring in both central and effector memory T cells. Moreover, decreased regulatory T cells occur in naïve regulatory T cells. The CD8^+^ T cells present impaired TLR activation responses, an increase in CD38 expression in the TEMRA subset, and decreased CD127^+^, indicating an immunosenescence phenotype of CD8^+^ T cells in CVID patients. However, TLR7/TLR8 agonists stimulated Th22/Tc22 cells. Activation with TLR2 and TLR7/8 agonists induced a polyfunctional response only in CD4^+^ CD38^−^ T cells. These data show a potential role for TLR ligands as adjuvants to stimulate adaptive T cell responses, and they provide evidence for a CD8^+^ T cell population that is unresponsive to innate stimuli in CVID.

The increased expression of the exhaustion marker PD-1^+^ in CD4^+^ T cells was confirmed as described [17], occurring in the TEM and TCM subsets in CVID patients despite long-term IVIg treatment. The duration of IVIg treatment in CVID patients was 9.0 [3–15] years. In parallel, an increased frequency of activated CD8^+^ CD127^−^ CD38^+^ T cells was detected in terminally differentiated CD8^+^ TEMRA. The activation and exhaustion markers in CVID have been associated with immune system aging [17]. In this context, our findings confirm immunosenescence features in CVID [21] but reveal that T cell exhaustion was related to the CD4^+^ TEM subset and T cell activation of the terminally differentiated T cells.

We also observed that exhaustion and activation markers in CD4^+^ and CD8^+^ T cells, respectively, do not seem to be associated with splenomegaly, a predominant clinical feature of CVID (43.7 % of selected patients). Tregs are crucial to control immune responses and limit persistent immune activation [25]. Stratifying CVID patients by splenomegaly showed an association with a low percentage of naïve Treg cells. Of naïve and peripherally induced Treg, only naïve Treg showed a decreased frequency in CVID. CVID patients with autoimmunity and/or splenomegaly had a reduced frequency of CD4^+^ CD25^hi^ Tregs, which was more accentuated in those with splenomegaly; additionally, they were associated with CD21^low^ B cell expansion [3]. A subgroup of CVID patients with CD21^low^ B cell expansion showed increased incidence of splenomegaly and autoimmune cytopenia [18]. In our CVID cohort, we found an autoimmune phenotype in 19 % of patients and splenomegaly in 25 % of patients. Our findings confirm the decrease in Tregs in CVID; despite long-term IgG replacement, this therapy could not control the naïve Treg cells in CVID patients with splenomegaly, suggesting that Tregs are essential to limit persistent immune activation. In our cohort of CVID patients, we found an unusual frequency of splenomegaly reaching 50 %, whereas evaluation of the whole cohort of patients resulted in an observed 25 % frequency of splenomegaly, as reported by Gathmann et al. [26].

Passive immunotherapy with IVIg in CVID can partially restore the CD4^+^ T cell compartment and reduce CD8^+^ T cell activation [17]. Moreover, the CD4^+^PD-1^+^ T cell exhaustion and functional impairment observed in CVID patients seems to be associated with bacterial translocation, and IVIg treatment decreases bacterial translocation, restoring CD4^+^ T cell functions [11]. Interestingly, bacteria-specific but not virus-specific CD4^+^ T cells express higher levels of PD-1 [11]. Curiously, we observed that the TLR4-mediated cytokine response by CD4^+^ T cells was unaltered in CVID patients (data not shown), whereas intracellular TLR3/Poly-RIG activation led to decreased IFN-γ, IL-17a and IL-22 responses by CD8^+^ T cells but not CD4^+^ T cells. In fact, the TLR3 pathway is of paramount importance to stimulate the secretion of type I IFNs through IRF3. TLR3 signaling defects are associated with specific susceptibility to herpes simplex virus type 1 encephalitis [27].

The investigation of TLR activation in CVID patients is primarily related to determining the effects among B cells. CVID patients show defective TLR7 and TLR9 signaling in B cells and pDCs [6]. These TLR defects in CVID are restricted because PBMCs stimulated with TLR ligands produce normal amounts of TNF-α, IL-6, and IL-12 [6]. In our CVID patients, activation via TLR7/TLR8 caused an in vitro expansion in circulating Th22/Tc22 cells. It is possible that TLR8 activation overcomes the TLR7 defects. CL097, an agonist of TLR7/TLR8, is an important adjuvant to stimulate the antiviral response [28, 29]. It is possible that the increased frequency of Th22/Tc22 cells in response to TLR7/8 in CVID contributes to mucosal protection. In fact, IL-22 at mucosal surfaces provides innate immune protection against bacterial and fungal infections and promotes inflammation and epithelial proliferation and repair [30, 31]. Sigmoid IL-22-producing T cells and Th22 cells are dramatically depleted during chronic HIV infection, and this depletion contributes to epithelial damage and microbial translocation [32]. It is important to stress that CVID patients are prone to inflammatory bowel disease and present lymphoid nodular hyperplasia throughout the gastrointestinal (GI) tract, which is clear evidence of chronic activation of the GI immune system. If the activation/exhaustion of T cells could be associated with bacterial translocation in CVID [11], Th22/Tc22 activation by TLR7/8 stimulation may be an interesting strategy to potentiate their functional response.

Despite the immunomodulatory role of TLR agonists in T cells, the CD4^+^/CD8^+^ T cell response to PMA/ionomycin was impaired in CVID. PMA/ionomycin-mediated activation of NFAT signaling may mimic full activation by TCR signaling and costimulation or anergy [33]. The involvement f Ca^2+^ T cell signaling in CVID has yet to be further investigated.

Although innate immunity may constitute the primary functions of TLRs, their role in T cells is less well known. The in vitro activation of PBMCs with TLR agonists may activate APCs and indirectly lead to T cell activation. However, TLRs can be expressed in T cells [34] and serve as costimulatory signals in T cell activation [35, 36]. TLR2 serves as a costimulatory receptor for antigen-specific T cell development and participates in the maintenance of T cell memory [14]. These findings suggest that pathogens may contribute directly to the perpetuation and activation of long-term T cell memory in both an antigen-dependent and independent manner.

Polyfunctionality is the ability to produce multiple cytokines, which has been associated with beneficial immune responses. As we observed the exhaustion/activated profiles of CD4^+^ T cells and CD8^+^ T cells in CVID, we next evaluated their ability to secrete several cytokines upon TLR activation. CD4^+^ and CD8^+^ T cells preserved their ability to secrete five TLR-induced cytokines in CVID, although CD8^+^ T cells were less polyfunctional than CD4^+^ T cells. Both extracellular (TLR2/Pam3CKS4) and intracellular TLR agonists (TLR7/8/CL097) induced CD4^+^ T cells to secrete three cytokines in CVID, including IL-17a, IL-22 and TNF. TLR2 stimulation drives human naïve and effector regulatory T cells into a Th17-like phenotype with reduced suppressive function [37]. Polyfunctionality was related to CD4^+^CD38^−^ T cells in response to TLR activation in CVID, whereas CD8^+^ T cells were unresponsive. These data suggest an altered CD8^+^ T cell response to TLR activation in CVID, in either a monofunctional and polyfunctional function.

## Conclusions

Our findings show that despite the long duration of IVIg replacement, CVID patients have altered responses to TLR activation, mainly among CD8^+^ T cells. Activation with TLR2 and TLR7/8 agonists induced a polyfunctional CD4^+^ T cell response, which was impaired in CD4^+^CD38^+^ T cells. These data show a potential role for TLR7/TLR8 ligands as adjuvants to stimulate adaptive T cell responses, and they provide evidence for a CD8^+^ T cell population that is unresponsive to innate stimuli in CVID.

## Additional files

10.1186/s12967-016-0900-2 Gating strategy for monofunctional CD4+ and CD8+ T cells. Initially, cells were selected from gates SSC-A and SSC-H (singlets) to exclude doublets; afterwards, we selected lymphocyte cells, live cells and CD3+ T cells, and the subsets CD4+ and CD8+ . Each cytokine was individually defined: IL-17a, IL-22, TNF, IL-10 and IFN-γ.
10.1186/s12967-016-0900-2 Gating strategy for Th22 and Tc22. The initial gate utilized was side scatter area (SSC-A) versus side scatter height (SSC-H) to exclude doublets. Next, lymphocytes were selected, dead cells were excluded with LIVE/DEAD staining, and CD3+ T cells were selected for CD4+ or CD8+ T cells, excluding IFN-γ and IL-17a, and were evaluated for IL-22 production.
10.1186/s12967-016-0900-2 Gating strategy for polyfunctional CD4+ and CD8+ T cells. The expression of each cytokine was evaluated in CD3+ T cells, followed by CD4+ T or CD8+ cells in the same combinations. Next, Boolean evaluation of several combinations of secreted cytokines was performed, and CD38 expression was assessed.
