# Supplementary figures and images for: Impaired CD8+ T cell responses upon Toll-like receptor activation in common variable immunodeficiency
Source: J Transl Med. 2016 May 17;14:138. doi: 10.1186/s12967-016-0900-2 (PMC4870753; doi:10.1186/s12967-016-0900-2)

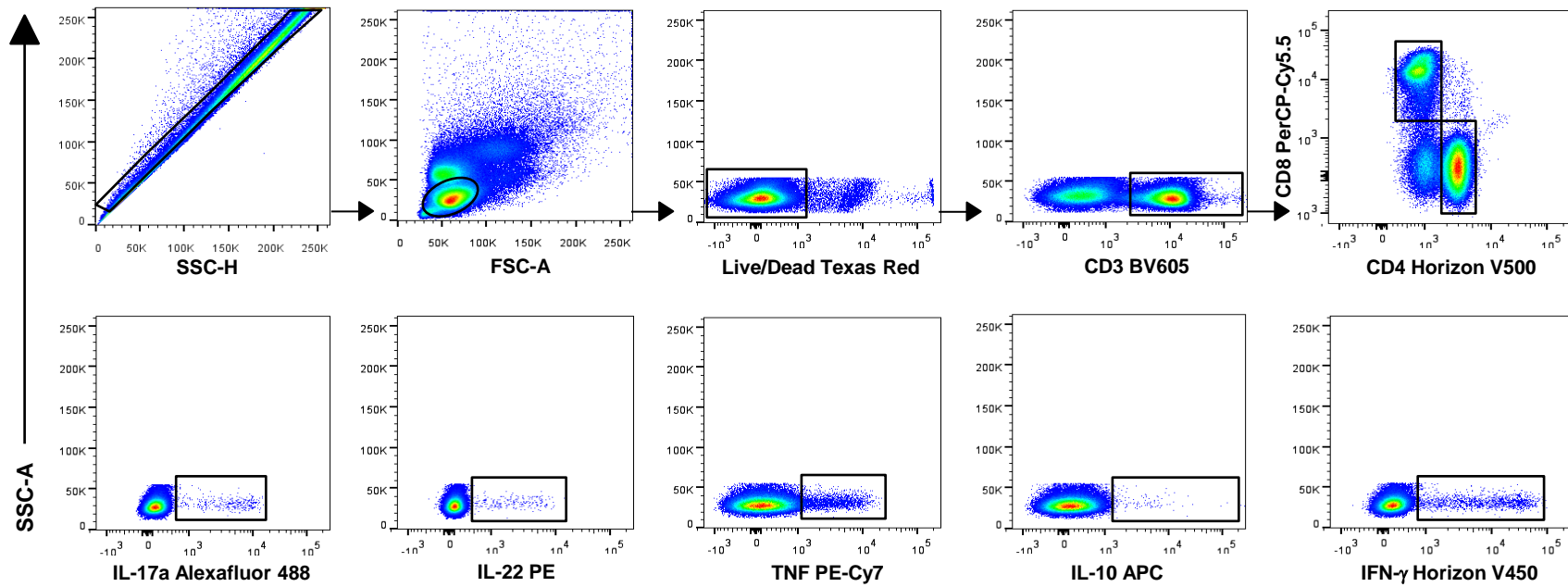

Supplement: Supplementary file 1 — 10.1186/s12967-016-0900-2 Gating strategy for monofunctional CD4+ and CD8+ T cells. Initially, cells were selected from gates SSC-A and SSC-H (singlets) to exclude doublets; afterwards, we selected lymphocyte cells, live cells and CD3+ T cells, and the subsets CD4+ and CD8+ . Each cytokine was individually defined: IL-17a, IL-22, TNF, IL-10 and IFN-γ. [file 12967_2016_900_MOESM1_ESM.pdf]

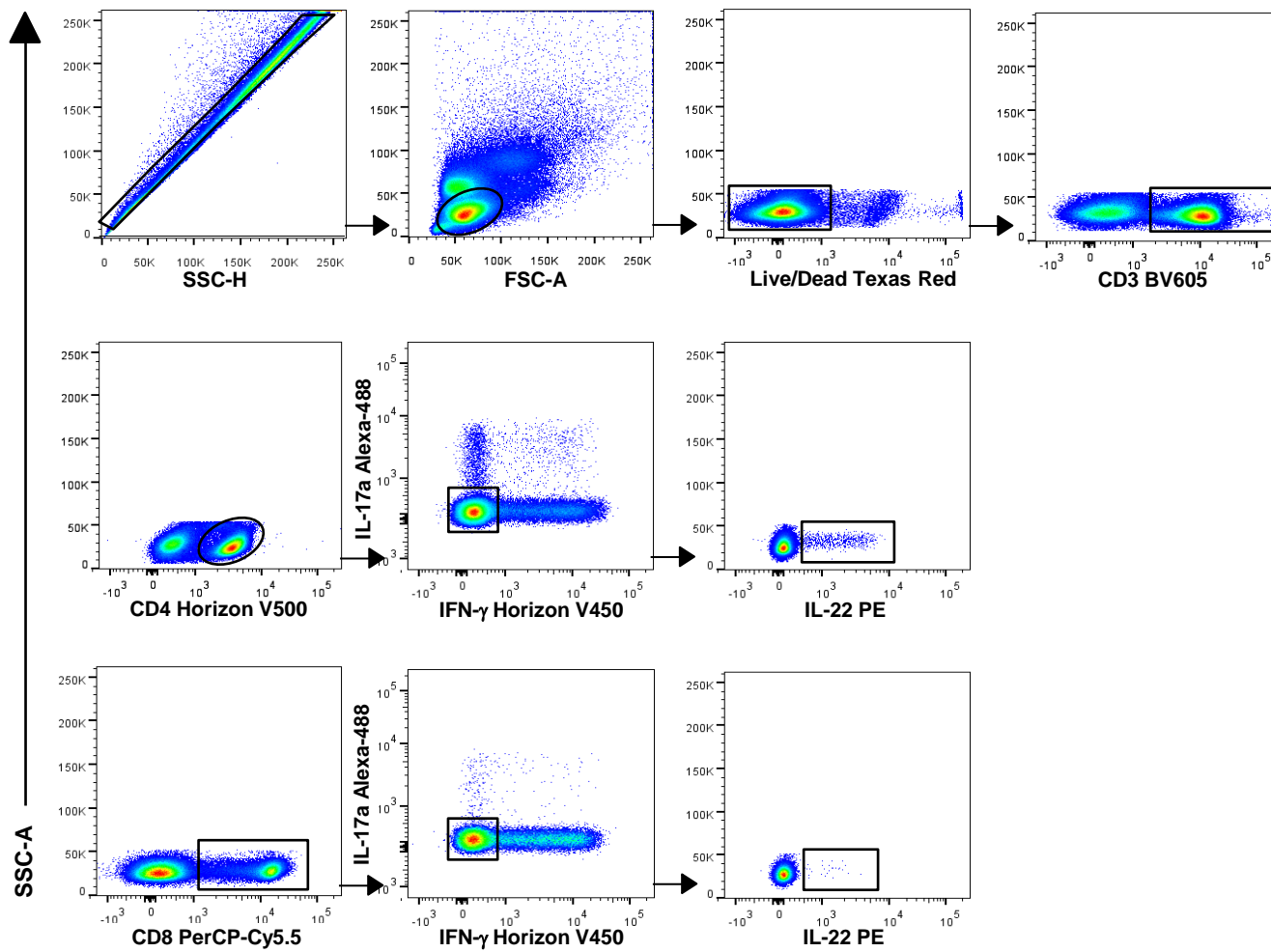

Supplement: Supplementary file 2 — 10.1186/s12967-016-0900-2 Gating strategy for Th22 and Tc22. The initial gate utilized was side scatter area (SSC-A) versus side scatter height (SSC-H) to exclude doublets. Next, lymphocytes were selected, dead cells were excluded with LIVE/DEAD staining, and CD3+ T cells were selected for CD4+ or CD8+ T cells, excluding IFN-γ and IL-17a, and were evaluated for IL-22 production. [file 12967_2016_900_MOESM2_ESM.pdf]

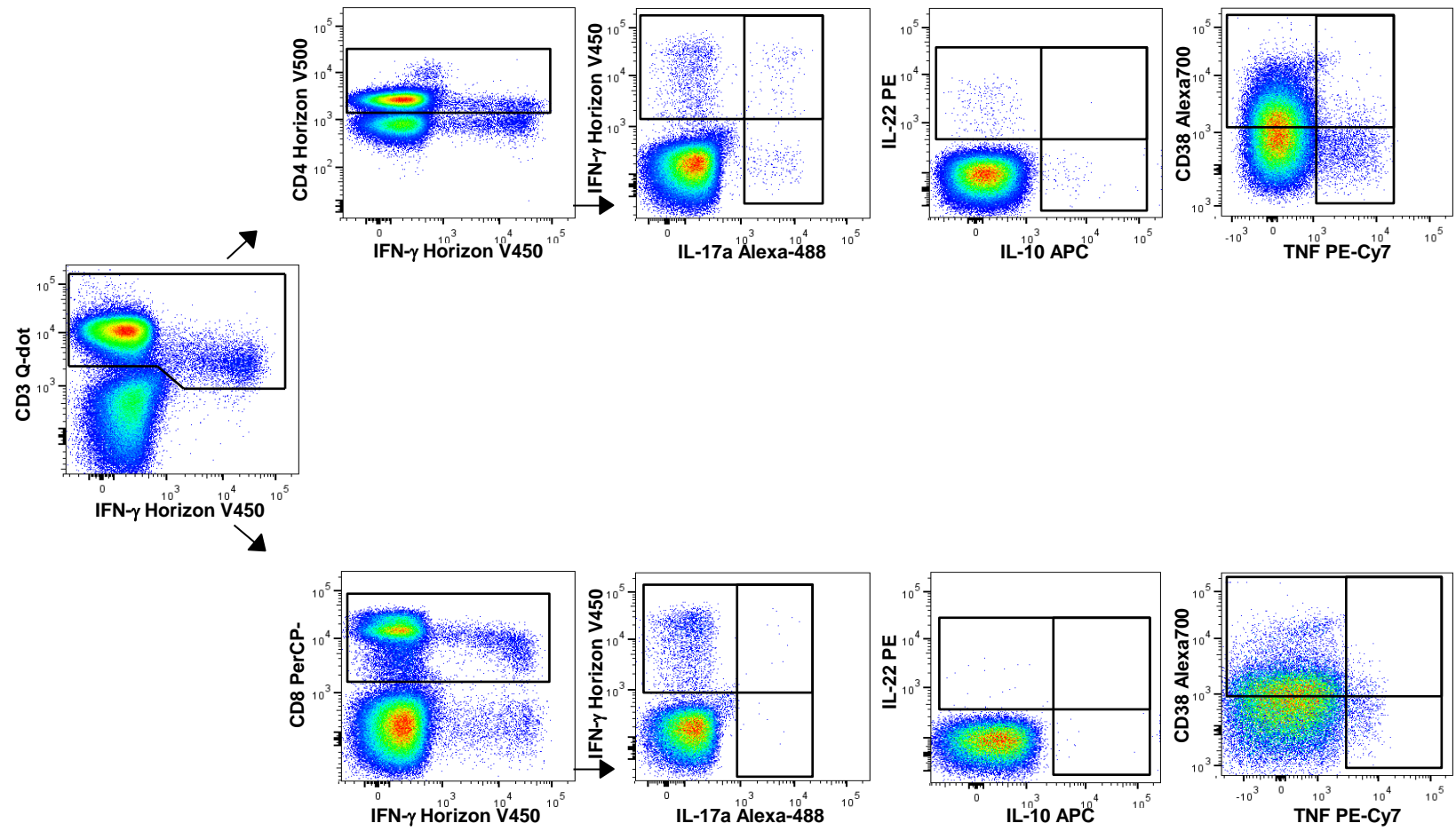

Supplement: Supplementary file 3 — 10.1186/s12967-016-0900-2 Gating strategy for polyfunctional CD4+ and CD8+ T cells. The expression of each cytokine was evaluated in CD3+ T cells, followed by CD4+ T or CD8+ cells in the same combinations. Next, Boolean evaluation of several combinations of secreted cytokines was performed, and CD38 expression was assessed. [file 12967_2016_900_MOESM3_ESM.pdf]
